# Using an upper extremity exoskeleton for semi-autonomous exercise during inpatient neurological rehabilitation- a pilot study

**DOI:** 10.1186/s12984-018-0415-6

**Published:** 2018-08-02

**Authors:** Imke Büsching, Aida Sehle, Jana Stürner, Joachim Liepert

**Affiliations:** 1 Department of Neurorehabilitation, Kliniken Schmieder, Zum Tafelholz 8, D- 78476 Allensbach, Germany; 2 Reha-Klinik Bellikon, Mutschellenstrasse 2, 5454 Bellikon, Switzerland

**Keywords:** Stroke, Inpatient rehabilitation, Robot-assisted upper limb training, Severe arm paresis, Virtual reality, Additional training

## Abstract

**Background:**

Motor deficits are the most common symptoms after stroke. There is some evidence that intensity and amount of exercises influence the degree of improvement of functions within the first 6 months after the injury.

The purpose of this pilot study was to evaluate the feasibility and acceptance of semi-autonomous exercises with an upper extremity exoskeleton in addition to an inpatient rehabilitation program. In addition, changes of motor functions were examined.

**Methods:**

Ten stroke patients with a severe upper extremity paresis were included. They were offered to perform a semi-autonomous training with a gravity-supported, computer-enhanced device (Armeo®Spring, Hocoma AG) six times per week for 4 weeks. Feasibility was evaluated by weekly structured interviews with patients and supervisors.

Motor functions were assessed before and after the training period using the Wolf Motor Function Test (WMFT). The Wilcoxon Signed Rank Test was used for assessing pre-post differences. The Pearson correlation co-efficient was used for correlating the number of completed sessions with the change in motor function. Acceptance of the device and the level of satisfaction with the training were determined by a questionnaire based on visual analogue scales.

**Results:**

Neither patients nor supervisors reported side effects. However, one patient had to be excluded from analysis because of transportation difficulties from the ward to the treatment facility. Therefore, analysis was based on nine patients. On average, 13.2 (55%) sessions were realized. WMFT results showed significant improvements of proximal arm functions. The number of sessions correlated with the degree of shoulder force improvement. Patients rated the exercises to be motivating, and enjoyable and would continue using the Armeo®Spring at home if they had the opportunity.

**Conclusion:**

Using an upper extremity exoskeleton for semi-autonomous training in an inpatient setting is feasible without side effects and is positively rated by the patients. It might further support the recovery of upper extremity function.

**Trial registration:**

The trial was retrospectively registered. Registration number ISRCTN42633681.

## Background

Stroke belongs to the most common causes of death and disability worldwide [1]. The prevalence increases continuously with age and affects approximately 7% of persons aged 70–79 years [2]. Motor deficits after stroke can be found in up to 82% of the patients [3], and 6 months after stroke, 65% still have difficulties to incorporate the affected upper extremity in activities of daily living [4]. Numerous rehabilitative strategies have been developed for improvement of motor functions [5]. One of these is the use of robot-assisted devices. A recent review concluded that electromechanical and robot-assisted arm training improved activities of daily living, arm function and arm muscle strength more than conventional therapies [6]. Another issue still open for discussion is the dose-response relationship. Some evidence is available that more movement practice results in better outcomes [7, 8]. It is recommended to increase exercise intensity by making the tasks more difficult and/or increasing the number of repetitions [9, 10]. Presumably, robot-assisted therapy is effective because it allows to deliver high-dosage and high-intensity training [11].

In our study, we were interested if technology-assisted exercises that are offered in addition to a conventional inpatient neurological rehabilitation program are accepted by the patients and if such an extra-training apart from the usual therapy schedule and without support by therapists is feasible. We included severely affected stroke patients without ability to use the upper extremity in activities of daily living. An exoskeleton that provides an adjustable arm support and allows gravity-supported and computer-enhanced arm exercises (ArmeoSpring) was chosen for the additional training [12]. Several studies using the ArmeoSpring device have already demonstrated improvements in motor functions, including increases of strength, reductions of spasticity and pain [13–17].

## Methods

### Patient group

Nine subacute patients (mean time since stroke = 9.6 weeks ± 3 weeks) and one chronic patient (time since stroke = 3,5 years) were included. The group consisted of seven male and three female patients with a mean age of 59.2 ± 12.6 years. In four of the patients the right hemi-body was affected and six were left-side affected. Patients were included after an ischemic (*n* = 4) as well as after a hemorrhagic stroke (*n* = 6).

One patient had to be excluded from data analysis because of recurrent transportation problems from his ward to the treatment room, thus precluding his possibility to participate in the exercises. Data analysis therefore was performed with nine patients.

### Inclusion criteria

The inclusion criteria for the study were that the patient suffered a first stroke with a severe upper limb paresis with a non-functional paretic hand. The patient should be able to give informed consent and to understand and follow all instructions. Inpatient rehabilitation should at least continue for 4 weeks after inclusion.

### Exclusion criteria

The exclusion criteria were psychiatric diseases, inability to give informed consent or to understand and follow the instructions e.g. because of dementia or aphasia. We also excluded patients with an upper limb pain > 3 (visual analogue scale) and a spasticity ≥2 (Modified Ashworth Scale). The reason for choosing these thresholds for pain and spasticity was that higher degrees of pain or spasticity might interfere with training and assessments.

The study was approved by the ethical committee of the University of Constance. Patients gave informed consent before entering the study.

### Assessments

Feasibility was explored by structured interviews with patients and supervisors once per week. In addition, both groups were asked to report any unexpected events immediately. The interview focused on side effects during the training session and any adverse event in association with the training.

In order to record the changes of arm and hand motor skills, the patients were tested at baseline and after the training period with the Wolf Motor Function Test (WMFT) [18]. The WMFT includes seven items for shoulder and arm movements, one item for shoulder-strength and nine items for hand and finger movements.The execution times for each item are added. If an item is not feasible for the patient, a time penalty of 120 s is given. The subtest of shoulder force measures how much weight the patient can lift with his affected arm from the table onto a little box in front of him by using weight cuffs which are fixed at the back of the hand.

The acceptance of the device and the level of satisfaction with the training were determined by seven questions (Table 2). The rating was performed with a visual analogue scale (VAS) [19]. The patients were asked to mark a position on a horizontal non-scaled line with a length of 10 cm. The left-sided end of the line represented the maximum negative response. The right-hand end of the line indicated the maximum positive response. For analysis the distance from the left-sided end of the line to where the patient had placed the mark was measured with a ruler. For each question the mean value of the nine patients was calculated. Furthermore, we asked the patients to judge the amount of training (too little – just enough – too much).

The number of realized training sessions (out of a maximum from 24 appointments) and the duration of each training session were recorded. Based on this, the percentage of utilization and the mean duration of the sessions were calculated.

After termination of the treatment period and analysis of utilization patients were contacted by telephone in order to explore why the patients had not realized all offered sessions. We performed a semi-structured interview. First, we asked them to provide reasons for non-participation. Then, we specifically asked for motivational aspects, fatigue, factors as having visitors, staying at home on a weekend day, transient illnesses. The latter issue was also derived from the patient’s chart.

### Training device

We used the Armeo®Spring exoskeleton (Hocoma AG, Zurich, Switzerland) which is a passive instrumented arm orthosis with a spring mechanism for adjustable arm weight support, combined with a training software program and a 3D workspace.

The exoskeleton gives weight support to the patient’s paretic arm and hand. The amount of weight support, the training workspace as well as the complexity of the virtual tasks can be individually chosen and adjusted to the patient’s active movement capacity. Movement parameters include muscle strength, active range of motion, movement velocity, coordination, and the ability to lift the arm against gravity.

In preparation of this study, Hocoma AG developed a self-training module within the Armeo®Control software which can be accessed via login passwort by the patient himself/herself, by a patient’s relative or by the training supervisor. Thus the patient is enabled to perform an individualized, semi-autonomous extra training without having to know the details of digital adjustment of the software and without the presence of skilled therapy staff. The choice and difficulty level of those individualized training tasks and games, however, can only be adjusted by skilled therapists.

The semi-autonomous training was supervised by persons without therapeutical education who had been instructed how to adjust the exoskeleton to the affected arm and how to login in case the patient was unable to enter in his/her password. Once this setup was finished the patients had to exercise on their own. One patient was accompanied by his wife on a regular basis, all other patients were rarely accompanied by a family member (once per week).

### Study design and procedure

All participants received the conventional, intense neurorehabilitation program with a multidisciplinary approach offered at our institution. The treatments addressing the upper extremity included occupational therapy, upper extremity circuit training, exercises with the SAEBOflex (an orthosis which supports finger extension mechanically by implementation of an extension spring that assists in re-opening the hand), bimanual co-ordination exercises, functional electrical stimulation.

Self-contained training with the Armeo®Spring device was possible on weekends and during early evenings. The room was open for 2 h per day. We recommended to perform exercises for 30 min per session. Thus, up to 3 patients could perform their training one after the other. It took 5 to 8 min to adjust the ArmeoSpring according to each patient’s individual needs. Therefore, a fourth patient would not have sufficient time for training within the given time window. The additional exercise opportunities were offered on 6 days per week for a period of 4 weeks. Thus, a maximum of 2 4 additional treatments was available,

After baseline evaluation, an occupational therapist (who was member of the research group) defined the initial setup of software parameters individually. In the same session, an individualized, 30 min training was compelled for the patient, The following day was defined as the start of the training period.

Further adjustments of the difficulty levels were done once a week according to the patient’s clinical development. In detail, the patient’s performance with the ArmeoSpring was assessed and more difficult programs were chosen if the patient showed a high performance level on the hitherto existing tasks. We carefully avoided excessive increases of task difficulty in order to prevent frustration [20].

### Statistical analysis

The statistical evaluation was carried out with IBM SPSS Statistics 24.

The “Wilcoxon Signed Rank Test”was used for assessment of pre-post differences. Correlations between the number of realized sessions and changes of motor performance were done with the Pearson correlation co-efficient. Statistical significance was set at *p* < 0,05.

## Results

### Utilization

On average, 13.2 appointments were used, corresponding to a percentage use of 55 ± 13.4%. The range of completed sessions was between 9 and 18, corresponding to a median of 61.9% with a range between 37.5 and 75%. The mean duration of each training session was 33 ± 8 min. The most frequent reason for omission of a session was being too fatigued by the regular training program (8 patients). Other reasons were: having visitors during the weekend (6 patients), spending one weekend day at home (5 patients), feeling not well due to a cold (3 patients), transient lack of motivation (2 patients), diarrhea (1 patient), low back pain (1 patient).

The number of conventional therapy sessions aimed at improving upper extremity function was on average 6.7 per week, corresponding to 3.78 ± 1.1 h per week and 15.1 ± 4.5 h for the 4 week period. With additional semi-autonomous training the number increased to 9.6 sessions per week, corresponding to 5.39 ± 1.4 h per week and 21.6 ± 5.6 h for the 4 week period.

The duration of inpatient rehabilitation ranged from 6 to 22 weeks with a mean of 12 ± 4.3 weeks. During this time, patients received a mean of 44.3 ± 16.4 h of therapies focusing on the upper extremity.

A total of 15 different PC games was offered. In 14 games, patients trained horizontal abductions and adductions (all patients), in 8 games shoulder extension and flexion were exercised (eight patients), in four games grasping and releasing were trained (four patients), in three games elbow flexion and extension were exercised (four patients), in two games supination and pronation were trained (two patients) and in one game internal and external shoulder rotation was trained (one patient). The amount of anti-gravity mechanical support provided by the ArmeoSpring was adjusted by the supervisors on a weekly basis and changed from strong to less support.

### Motor assessments (Table 1)

**Table 1:** shows WMFT results before (pre) and after (post) the 4 week period. SD, standard deviation

WMFT items	pre (Mean value +/− SD)	post (Mean value +/− SD)	Significance
All items (15)	87.4 +/− 18.2 (seconds)	73.0 +/− 27.1 (seconds)	*p* = 0.066
Including the items for hand and arm			
Seven Items	51.4 +/− 37.0 (seconds)	36.0 +/− 35.0 (seconds)	*p* = 0.038
Including the items for shoulder and arm			
One Item	0,6 kg +/− 0.9 (kilogram)	1.6 +/− 1.5 (kilogram)	*p* = 0.027
Shoulder-force			

### Correlations

The correlation between the number of used appointments and pre-post differences was not significant for the 15 WMFT items; however, the co-efficient indicated a trend.

(*r* = 0.645; *p* = 0.061). No significant correlation was found for the seven WMFT items of shoulder and arm functions (*r* = 0.474; *p* = 0.189).

**Fig. 1:**
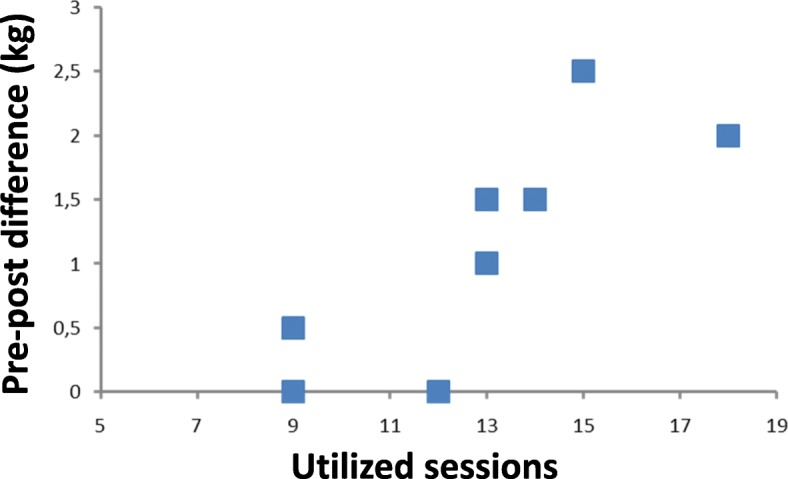
The difference in weight lifting (post treatment minus pre treatment result), expressed in kg, was correlated with the number of sessions each patient participated in

A significant correlation could be demonstrated for the WMFT subtest “weight lifting” which basically measures shoulder force (*r* = 0.834; *p* = 0.005) (Fig. 1).

### Acceptance by the patients (Table 2)

Results show that patients rated the exercises to be motivating, enjoyable and easy to understand. The participants also stated that they were satisfied with their own performance of the exercises. They indicated that they likely would continue the training in their home environment if there was an Armeo®Spring device close to their home.

**Table 2:** shows the questions and the results. MV, mean value; SD, standard deviation

Questions	MV	SD
1.) How strongly did the training with the Armeo®Spring help to improve your arm functions? (0 = not at all; 10 = very strongly)	5.42	3.58
2.) How much fun did you have during the exercises? (0 = no fun at all; 10 = a lot of fun)	7.04	3.22
3.) How motivating were the exercises for you? (0 = not motivating at all; 10 = very motivating)	7.10	2.51
4.) How simple did you find it to insert your arm into the device before starting the exercises? (0 = very difficult; 10 = very easy)	5.5	2,89
5.) How easy was it to understand the exercises? (0 = very poorly understandable; 10 = very well understandable)	8.67	1.75
6.) How good was your performance? (0 = very bad; 10 = very good)	7.29	1.44
7.) If there was an Armeo®Spring device close to your home, how likely would it be that you continue the exercises there? (0 = very unlikely; 10 = most likely)	8.39	3,19

The effect of the additional training at the Armeo®Spring was estimated by the patients in the middle section of the VAS scale. Furthermore, it was found that inserting and attaching the arm into the exoskeleton was classified as feasible but not simple.

Patients suffering from neglect symptoms might have difficulties to correctly identify the middle of a line. Therefore, we re-analyzed the data by subdividing the group into patients with (*n* = 4) and without neglect (*n* = 6). The allocation of patients to the group with or the group without neglect was based on detailed neuropsychological tests that detected neglect symptoms even without neglect being relevant in activities of daily living. If neglect had an impact on our method of data acquisition, neglect patients would have achieved higher numbers. The results between the two groups did not differ. We consider this as indirect evidence that there was no “mislocalization” when marking the position on the line. Moreover, during exercising with the ArmeoSpring, there was no indication of less attention for the left part of the screen.

Eight patients stated that the amount of used appointments was just right for them. One patient mentioned that she would have liked to practice more. Indeed, she had used most of the appointments (18/24). She missed two of the appointments due to a cold and the other four appointments due to weekend days spent at home.

## Discussion

### Feasibility

This study demonstrates that semi-autonomous training with a exoskeleton is feasible for stroke patients with severe upper extremity paresis but it also indicates some limitations. To our knowledge, this is the first study to explore such a training within an inpatient rehabilitation period. Other groups have demonstrated that technology-assisted self-administered training is feasible at the patient’s home [21–24].

During the treatment period, no adverse effects occurred. None of the patients aborted the study due to problems with the device or the exercises. Subjective ratings indicated an overall satisfaction with the additional treatment option. After the treatment period some motor functions were significantly improved. However, due to the lack of a control group, it is impossible to ascribe these improvements to the additional exercises.

A major limitation of feasibility was the patient transportation from the ward to the treatment room and back. In order to keep additional involvement of staff as low as possible, we deliberately decided not to organize this transport by employees of the hospital. As a consequence, one of the patients had to be excluded from the study because he had been unable to attend the offered appointments due to transportation difficulties. This limitation is, of course, restricted to severely affected patients. Patients with minor to moderate motor deficits would not have had problems with reaching the treatment facilities. For future studies or the implementation of such an additional training as an adjunct to conventional in-patient rehabilitation the question of transportation needs to be solved.

Fortunately, realisation of therapy was easy and without difficulty. The supervising persons were able to use the Armeo®Spring exoskeleton after a brief introduction of 3 h. They did not report problems with handling of device or patients. Thus, the staff requirements for such an additional training are limited.

On average, the patients took advantage of only about half of the offered training appointments, with a considerable range of usage from a minimum of nine and a maximum of 18 used appointments. The reasons for not participating in all offered appointments varied. Fatigue induced by the regular inpatient exercise program was the most important issue. This suggests that for more severely affected patients the capacity for increasing the amount of therapies during an inpatient rehabilitation is limited. Our results suggest that 3 to 4 additional exercise sessions per week are well accepted but that 6 additional sessions per week are not feasible for all patients.

### Motor functions

After 4 weeks of additional training significant improvements of motor functions were found for shoulder force and for those items of the WMFT that evaluate proximal arm functions.. Since most patients were in the subacute phase after stroke and all participated in an inpatient rehabilitation program and due to the lack of a control group it is not possible to ascribe these improvements to the additional exercises. However, notably, motor improvements were most prominent for those functions that are typically trained with the Armeo®Spring. In addition, we found a correlation between the number of extra training sessions and the degree of shoulder force improvement. This may be interpreted as an indirect indication that additional exercises contributed to improvements of proximal arm functions. However, in order to assess the effects of additional training a randomized controlled trial is required. Several studies using the ArmeoSpring device have already demonstrated improvements in motor functions. Chan et al. [13] found an improvement of vertical control in subacute stroke patients. Colomer et al. [14] administered 36 sessions of ArmeoSpring training to chronic stroke patients and described improvements of function scales (Fugl Meyer Assessment, Motricity Index) and activity scales (e.g., Manual Function Test, Wolf Motor Function Test). In a group of subacute stroke patients Armeo®Spring exercises were associated with enhanced maximum range of motion for shoulder abduction/adduction [15]. Taveggia et al. [16] reported similar improvements of strength, spasticity and pain in the patient group that received Armeo®Spring exercises and the control group that received conventional physiotherapy. However, at follow up (6 weeks later) the robot-treated group showed further improvements of strength and pain. Grimm et al. [17] combined Armeo®Spring training with adaptive closed-loop feedback in virtual reality and reported beneficial effects in severely affected chronic stroke patients. Positive results have also been described with other devices as the resonating arm exerciser [25]. Possibly, improvements induced by robotic therapy are distinct from those obtained by conventional therapy, thus making these two approaches complementary [26].

### Self ratings

Participants uttered a high acceptance of the training, ranking the exercises to be well understandable, motivating and enjoyable, with mean values between 8.67 and 7.04. The high value in stating the will to continue the training in an ambulatory setting emphasizes the motivation of the participants. Other research groups have published similar results indicating a higher level of acceptance for robotic therapies with gaming elements (e.g. [27]).

The clamping of the arm into the exoskeleton was classified as feasible but not really simple. The patients judged their own performance in the exercises to be good. The answer with the lowest value and the highest standard deviation occurred when asking how strongly the training helped to improve their arm functions. Presumably, this reluctant answer is due to the fact that despite improvements of proximal arm functions it remained difficult to use the arm in activities of daily living because of a persistently impaired hand function.

### Limitations

This study was designed as a pilot study. The significance is limited due to the small sample size, the lack of a control group and some heterogeneity of the group, since one of the patients was in the chronic phase after stroke.

## Conclusions

Semi-autonomous training with an upper extremity exoskeleton in addition to standard inpatient therapy was feasible and well-accepted by stroke patients with a severe upper extremity paresis. Preliminary data suggest that motor function improvement might be associated with the amount of additional exercises. The WMFT was sensitive enough to display the improvements. However, patients did not make use of all appointments that were offered, indicating that *daily* additional training is less accepted or may not be feasible.

## Abbreviations

MV: Mean value
SD: Standard deviation
VAS: Visual analogue scale
WMFT: Wolf motor function test
